# (A)symmetry during gait initiation in people with Parkinson’s disease: A motor and cortical activity exploratory study

**DOI:** 10.3389/fnagi.2023.1142540

**Published:** 2023-04-17

**Authors:** Murilo Henrique Faria, Lucas Simieli, Shirley Rietdyk, Tiago Penedo, Felipe Balistieri Santinelli, Fabio Augusto Barbieri

**Affiliations:** ^1^Human Movement Research Laboratory (MOVI-LAB), School of Sciences, Department of Physical Education, São Paulo State University (Unesp), Bauru, São Paulo, Brazil; ^2^Department of Health and Kinesiology, Purdue University, West Lafayette, IN, United States; ^3^REVAL Rehabilitation Research Center, Faculty of Rehabilitation Sciences, Hasselt University, Hasselt, Belgium

**Keywords:** asymmetry, Parkinson’s disease, cortical activity, gait initiation, obstacle avoidance

## Abstract

**Background:**

Gait asymmetry and deficits in gait initiation (GI) are among the most disabling symptoms in people with Parkinson’s disease (PwPD). Understanding if PwPD with reduced asymmetry during GI have higher asymmetry in cortical activity may provide support for an adaptive mechanism to improve GI, particularly in the presence of an obstacle.

**Objective:**

This study quantified the asymmetry of anticipatory postural adjustments (APAs), stepping parameters and cortical activity during GI, and tested if the presence of an obstacle regulates asymmetry in PwPD.

**Methods:**

Sixteen PwPD and 16 control group (CG) performed 20-trials in two conditions: unobstructed and obstructed GI with right and left limbs. We measured, through symmetry index, (i) motor parameters: APAs and stepping, and (ii) cortical activity: the PSD of the frontal, sensorimotor and occipital areas during APA, STEP-I (moment of heel-off of the leading foot in the GI until the heel contact of the same foot); and STEP-II (moment of the heel-off of the trailing foot in the GI until the heel contact of the same foot) phases.

**Results:**

Parkinson’s disease showed higher asymmetry in cortical activity during APA, STEP-I and STEP-II phases and step velocity (STEP-II phase) during unobstructed GI than CG. However, unexpectedly, PwPD reduced the level of asymmetry of anterior–posterior displacement (*p* < 0.01) and medial-lateral velocity (*p* < 0.05) of the APAs. Also, when an obstacle was in place, PwPD showed higher APAs asymmetry (medial-lateral velocity: *p* < 0.002), with reduced and increased asymmetry of the cortical activity during APA and STEP-I phases, respectively.

**Conclusion:**

Parkinson’s disease were not motor asymmetric during GI, indicating that higher cortical activity asymmetry can be interpreted as an adaptive behavior to reduce motor asymmetry. In addition, the presence of obstacle did not regulate motor asymmetry during GI in PwPD.

## 1. Introduction

Gait initiation (GI) difficulties (Delval et al., 2014) and gait asymmetry (Barbieri et al., 2018a,b; Orcioli-Silva et al., 2020) are among the most incapacitating symptoms present in Parkinson’s disease. GI difficulties result from slower and smaller center of pressure (CoP) movement, which is influenced by reduced production of medial-lateral and anterior–posterior force (Rogers et al., 2011; Delval et al., 2014; Boonstra et al., 2016) (Simieli, 2021, under review)^1^, resulting in a shortened first step when initiating gait in people with Parkinson’s disease (PwPD; Simieli, 2021, under review, see footnote 1). Inhibitory alterations in the connection of basal ganglia with cortex and brainstem, specially non-dopaminergic system (e.g., pedunculopontine and cuneiform nucleus), explain motor impairments during GI in Parkinson’s disease (Delval et al., 2014). PwPD with GI difficulties present disrupted cortical activity (movement-related potentials) compared to those not experiencing this symptom (Shoushtarian et al., 2011). Gait asymmetry is caused by asymmetrical degeneration of both the dopaminergic and non-dopaminergic neural systems in Parkinson’s disease (Riederer and Sian-Hülsmann, 2012; Claassen et al., 2016), showing asymmetrical activity in motor cortex (Heinrichs-Graham et al., 2017). Also, cortical beta band synchronization, which is related to motor area activation or processing of information (Chung et al., 2017), is lower in the most affected limb (Tamás et al., 2006). However, it is unclear how motor and cortical activity behavior affects GI, which remains to be characterized.

GI asymmetry is commonly observed in different populations. For example, when people with hemiparesis initiate gait with the non-affected limb, a higher degree of uncertainty was observed due to the weakness of the trailing limb (affected limb), resulting in larger CoP oscillation in the medial-lateral direction (symmetric index: 32.5%), reduced relative swing time (symmetric index: 46.6%) and step length (symmetric index: 14.9%; Hesse et al., 1997). Also, individuals with severe unilateral knee arthritis (Viton et al., 2000) and hemiplegic people (Bensoussan et al., 2006) showed a reduced single support time (symmetric index: 2.7 and 46.6%, respectively) and forward propulsion (symmetric index: 11.6 and 88.1%, respectively) when they initiate the gait with non-affected limb. Finally, young adults without disability also show asymmetric behavior during GI – when the preferred limb was used for GI, the duration of anticipatory postural adjustments (APAs; symmetric index: 7.1%), and the displacement (symmetric index: 16.3%) and the velocity (symmetric index = 18.4%) of the CoP was larger, and ground reaction force was reduced compared to when non-preferred limb was used (Yiou and Do, 2010). As seen in other disease populations and considering to the motor asymmetry in Parkinson’s disease, it is expected that PwPD will increase motor asymmetry during GI.

To deal with these GI difficulties and a possible asymmetry during this task, compensatory strategies can be used. Previous studies reported that an attentional stimulus—visual cue—modulate APAs and cortical activation during GI in healthy adults (Tard et al., 2013; Braquet et al., 2020), improving event-related desynchronization in alpha and beta bands, reaction time and duration of the preparation phase when attention is focused on a target stimulus (Tard et al., 2013; Braquet et al., 2020). Obstacles (up to 15 cm in height) act as a visual cue during GI for PwPD. Recently our group showed that PwPD used the obstacle during GI as a trigger, generating visual stimulus that improved motor programming while offsetting the mechanical challenge imposed by the obstacle (Simieli, 2021, under review, see footnote 1). Maybe as GI requires more attentional resources than steady-state gait (Suzuki et al., 2004, 2008), the presence of an obstacle optimizes cognitive resources, modifying cortical activity, mainly in level of frontal activation (Maidan et al., 2016), and enhancing GI. Also, visual cues facilitate a compensatory shift to goal-directed control of movement, which is more robust to deterioration in Parkinson’s disease (Drucker et al., 2019), helping to bypass the impaired basal ganglia and improving the frontal activity (Beeler, 2011; Beeler et al., 2013). A more conscious motor control strategy reduces gait asymmetry during obstacle circumvention phase in PwPD (Corradini et al., 2022). The obstacle seems to be used as a guide in the avoidance phase, such as a visual reference to refine adjustments for positioning their feet to the obstacle, and the individual’s motor behavior to a safe performance (Corradini et al., 2022). A same compensatory effect could occur during GI with obstacle avoidance, reducing (regulating) asymmetry.

Therefore, this first exploratory study has two purposes. First, we will quantify the asymmetry of APAs, stepping parameters and cortical activity during GI in PwPD. Second, we will determine if the presence of an obstacle during GI alters the asymmetry of the parameters in PwPD. We hypothesize that PwPD would show higher asymmetry of APAs, stepping parameters and cortical activity during GI compared to neurologically healthy individuals. Specifically, we predict a higher asymmetry in the cortical activity in PwPD due to impaired neural processing (Heinrichs-Graham et al., 2014a; Stegemöller et al., 2016). This hypothesis is based on the observations that older adults reduce asymmetry in cortical activity (Hill et al., 2020; Knights et al., 2021) and PwPD show asymmetrical degeneration. A higher asymmetry may be characterized as adaptive or maladaptive change of motor control (Peterson and Fling, 2018; Berenguer-Rocha et al., 2020; Knights et al., 2021) of the neural systems (Riederer and Sian-Hülsmann, 2012; Claassen et al., 2016). Considering that the obstacle can guide GI and improve motor and brain control (Maidan et al., 2016; Corradini et al., 2022), we hypothesize that when an obstacle will be in place, the asymmetry of APAs and cortical activity parameters would reduce, regulating asymmetry, especially in PwPD vs. neurologically healthy people.

## 2. Method

### 2.1. Participants and clinical assessment

The study included 16 PwPD and 16 neurologically healthy people (CG). The number of participants was based on a power analysis using an alpha level of 0.05, effect size of 0.95 and a power of 95%, and data on APAs (CoP displacement) from Delval and colleagues (Delval et al., 2014; G-power)—the analysis indicated a minimum number of thirteen people in each group. Participants from both groups were recruited from the city of Bauru-SP, Brazil, and region through contact with doctors, physical activity groups, movement disorders centers, etc.

Inclusion criteria for PwPD: (i) diagnosis from a neurologist indicating the presence of Parkinson’s disease determined by the London’s Brain Bank (Hughes et al., 1992), (ii) stage 3 or less on the Hoehn & Yahr scale (Goetz et al., 2004) and iii) be under drug Parkinson’s disease treatment for more than 3-months. The following exclusion criteria were established for both groups: age below 50 years, cognitive decline assessed through the Mini Mental State Examination—MMSE [score below 24pts (Brucki et al., 2003) corrected according to the years of schooling], and orthopedic (such prothesis), vision (assessed through the Snellen chart test (Holladay, 2004) or vestibular (such dizziness) problems that made it impossible to carry out the experimental protocol. To assess motor severity of Parkinson’s disease the Unified Parkinson’s Disease Rating Scale—UPDRS-III—was applied (Goetz et al., 2007).

All participants provided their consent for the study approved by the Ethical Committee of School of Science at UNESP (CAAE #56031316.9.0000.5398). The PwPD were assessed in the “ON” state of Parkinson’s disease medication. To determine the lower-limb preference of the CG, the participants were asked to kick a ball, with the limb used to kick considered as the preferred (Barbieri et al., 2018a,b). For the PwPD, the most affected limb was determined through items 20–23 and 25–26 of UPDRS-III. The value of the right limb was subtracted from the value of the left limb in each item (Barbieri et al., 2018b). If this calculation resulted in a positive or negative value, the most affected limb was the right or left limb, respectively.

### 2.2. Protocol

**Figure 1:**
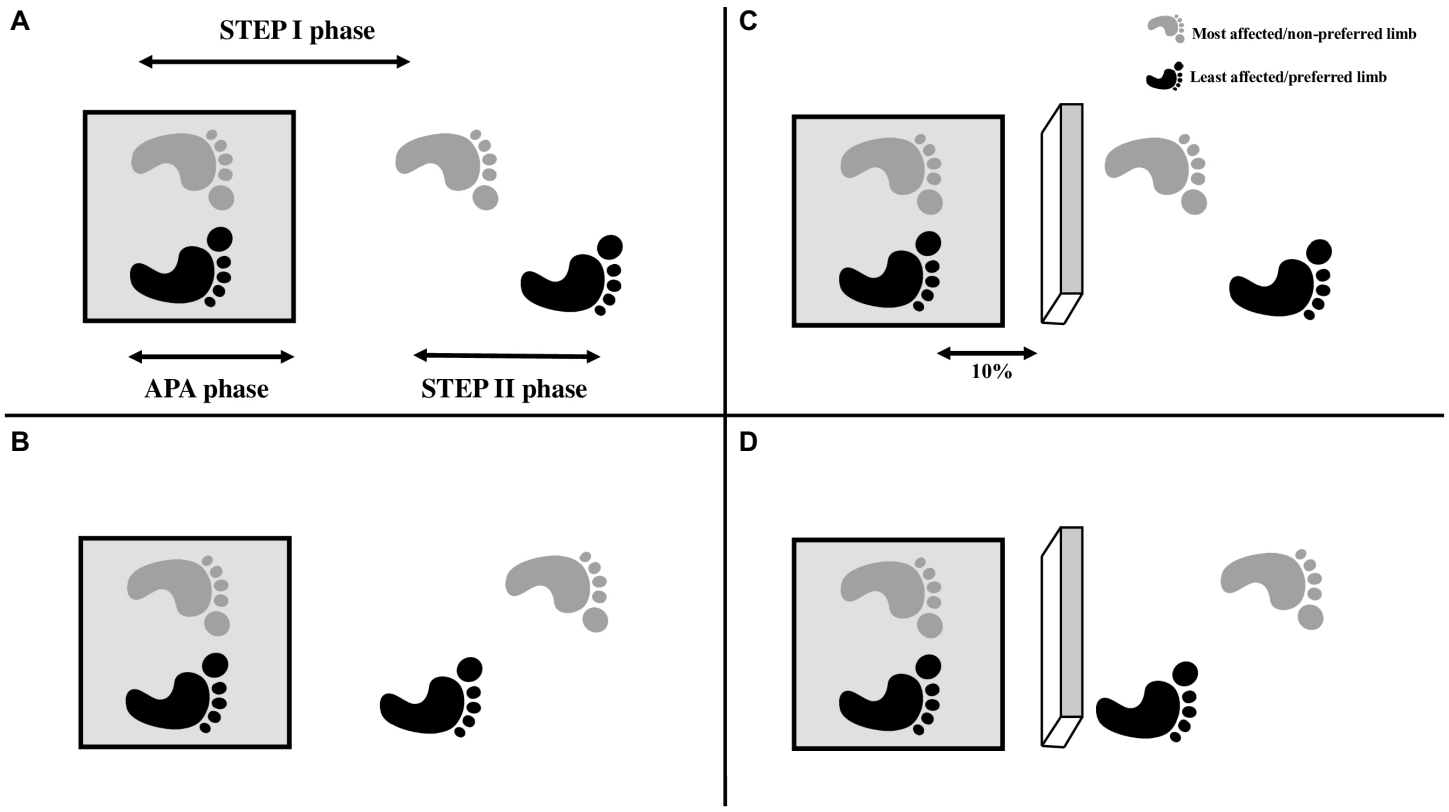
Exemplification of the conditions of the GI: **(A)** GI with the most affected/not preferred limb; **(B)** GI with the least affected limb/preferred limb; **(C)** Obstructed GI with the most affected/not preferred limb; **(D)** Obstructed GI with the least affected/preferred lower limb. The feet were positioned on the force plate (gray square). The arrows in the Figure A represent the phases of GI analyzed: APA phase—defined as the onset of changes in the CoP that occur before any foot apparent movement, based on the displacement and velocity of the heel marker; STEP I phase—the period corresponding to the step performed with the leading foot; STEP II phase—the period corresponding to the step performed with the trailing foot. The 10% in the Figure C represent the distance between participant’s feet and obstacle (10% of the participant’s height).

Each participant performed 20 GI trials in two conditions: unobstructed and obstructed (with obstacle avoidance; Figure 1). In each condition, the individuals performed five trials of GI using each limb. The participants were instructed previously which foot they should use to perform the GI. Also, the participants were instructed to keep their eyes closed until the command to start the trial, and walk until the end of walkway (~10 m). For the conditions in which the obstacle (15 cm high × 60 cm wide × 5 cm long) was present, the obstacle was positioned in front of participant’s feet at a distance equivalent to 10% of the participant’s height. The participants were instructed to avoid contact with the obstacle. The order of the conditions was randomized by blocks.

### 2.3. Equipment

A force plate (AMTI®, 200 Hz) was used to record CoP and, offline, to define the timing of the APAs. The kinematic parameters of 39 reflective passive markers, positioned according to the Full Body Plug-in-Gait model (Vicon Motion System®), were acquired by 10 infrared Vicon Motion System® cameras (200 Hz). The cortical activity was recorded by an electroencephalogram (EEG) equipment and was performed in accordance with the procedures suggested by the EEG manufacturer (eego™sports, ANT Neuro, Enschede, Netherlands, 1,024 Hz). A cap with 64 active electrodes, connected to an amplifier, was used, following the 10–10 International system electrode placement. The impedance remained below 10 KΩ on the electrodes.

### 2.4. Data analysis

The GI analysis was divided in three phases (Figure 1): (1) APA phase—defined as the onset of changes in the CoP that occur before any foot apparent movement, based on the displacement and velocity of the heel marker. APAs started with the initial movement of the CoP and ended when the CoP was in the most posterior and lateral in direction towards the stance leg (Nocera et al., 2013); (2) STEP I—defined as the moment of heel-off of the leading foot in the GI until the heel contact of the same foot; and (3) STEP II—defined as the moment of the heel-off of the trailing foot in the GI until the heel contact of the same foot.

The CoP data was filtered by a 4th order low-pass Butterworth filter with a cut-off frequency of 10 Hz (Nocera et al., 2013; Simieli, 2021, under review, see footnote 1). Five APAs parameters were calculated (normalized by the individual’s weight): displacement and velocity from the anterior–posterior and medial-lateral towards the swing leg, and peak of vertical force.

The heel and 2nd metatarsal markers were used to calculate stepping parameters. They were filtered by a 2nd order low-pass Butterworth filter with a cut-off frequency of 6 Hz. Step length, duration and velocity were calculated for both steps (STEP I and II phases).

The open-source software EEGlab (Delorme et al., 2011) was used for the EEG data analysis. First, the data from the 64 electrodes were imported into the EEGlab, and the position of the electrodes was defined (Delorme et al., 2011). The data was filtered with a band-pass filter (1-75 Hz), and a visual inspection was performed to remove highly apparent outliers (Whittier et al., 2020; Santinelli et al., 2021). Channels with an amplitude higher than 400 μV and channels with a value higher than four standard deviations from the mean were also removed (Santinelli et al., 2021). A down-sample was performed to 512 Hz and the data was re-referenced to the common average (Whittier et al., 2020). The Independent Component Analysis was performed to remove artifacts such as blinking and eye movement, muscle component and other potential artifacts (Radüntz et al., 2015). The average power spectral density analysis (PSD) was then performed in the following frequency bands, separately for each GI phase: *θ*: 4–7 Hz; *α*: 8–12 Hz; *β*: 13–30 Hz; *γ*: 31–50 Hz. The electrode data were grouped according to the following regions of interest: prefrontal cortex, sensorimotor cortex and occipital cortex (Tropini et al., 2011). The cortical activity data from one individual of the CG was excluded of the analysis due to data noise. To exclude the possibility of divergence when processing the EEG signals, only one researcher performed all procedures (Santinelli et al., 2021).

For the APAs, stepping, and cortical activity parameters, the symmetry index (Plotnik et al., 2005) was used to analyze the (A)symmetry between the conditions at the GI performed with the most/least affected limb for the PwDP, and preferred/non-preferred limb for the CG (Beretta et al., 2015; Barbieri et al., 2016). The symmetry index was calculated using the equation 1.


$$Symmetryindex=\left|\ln\;\left(\frac{\begin{array}{l} mostaffectedlimbornon\\ preferredlimb \end{array}}{\begin{array}{l} leastaffectedlimbor\\ preferredlimb \end{array}}\right)\right|X\;100$$

Where ln is the natural logarithm.

### 2.5. Statistical analysis

The level of significance was maintained at 0.05 for all parameters analyzed and SPSS 22.0 software (SPSS, Inc.) was used. The Shapiro–Wilk and Levene tests was used to check the data normality and homogeneity of the variances, respectively. An independent t-test was performed on anthropometric measures across groups. In addition, the Mann–Whitney *U*-test was used in order to compare the MMSE. To answer the first purpose of the study (to quantify asymmetry in GI for PwPD vs. controls in the unobstructed walkway), three one-way ANOVAs (PwPD vs. CG) were performed separately for the GI phases (APA, STEP I and STEP II phases) for the symmetry index of the APAs, stepping and cortical activity parameters only for unobstructed condition. To answer the second purpose of the study (to determine the impact of obstacle avoidance on the asymmetry in GI for PwPD), three two-way ANOVAs (group by GI condition: unobstructed vs. obstructed), for each GI phase, with repeated measures for the GI condition, were performed. Tukey’s post-hoc tests were performed when the ANOVA presented significance effect. Partial eta-squared (*η*^2^) was reported and used as effect size and interpreted as small (>0.01), moderate (>0.06) or large (>0.14) effect (Cohen et al., 2011).

## 3. Results

In Table 1 the characteristics of the participants are presented. The CG presented higher performance on cognition test than PwPD (*U* = 44.00; *z* = −3.26; *p* < 0.001). The groups were similar for age, body mass and height (*p* > 0.05). All PwPDs had included dopaminergic medication (levodopa) pharmacological treatment. Also, the participants were under treatment with other drugs: dopaminergic agonists (*n* = 8), amantadine (*n* = 4) and monoamine oxidase B (*n* = 1).

**Table 1:** Demographic and clinical characteristics of the participants.

Group	ID	Sex	Age (years)	Height (m)	Body mass (kg)	MMSE (pts)	Disease duration (years)	UPDRS III (score)	H&Y (score)	MA/NP limb	Daily levodopa (mg)
PwPD	PD01	M	66	1.71	71.6	25	1	17	3	L	400
	PD02	M	52	1.67	62.7	27	11	14	2	R	500
	PD03	F	68	1.59	68.2	26	7	39	2	L	50
	PD04	M	65	1.75	70.6	28	11	17	2	R	800
	PD05	F	63	1.60	67.3	27	3	31	1.5	L	400
	PD06	F	55	1.50	70.4	28	9	26	2	L	200
	PD07	M	84	1.61	66.4	26	5	33	2	R	200
	PD08	F	76	1.54	80.3	27	2	28	2	R	250
	PD09	F	68	1.58	76.9	28	4	24	2	L	400
	PD10	F	71	1.55	65.7	25	2	32	2	L	300
	PD11	M	59	1.62	69.0	25	8	28	1	L	400
	PD12	M	55	1.77	79.3	29	2	17	2.5	L	100
	PD13	F	67	1.57	46.5	28	7	10	1.5	L	600
	PD14	M	75	1.74	85.9	27	6	15	1	R	500
	PD15	M	62	1.75	84.5	29	3	28	2	R	200
	PD16	F	72	1.50	58.1	26	3	20	2	R	1,000
Mean ± SD		8F/8M	66 ± 8	1.64 ± 0.09	68.3 ± 9.7	26.9 ± 1.3	5.2 ± 3.2	23.2 ± 8.2	1.9 ± 0.4	7R/9 l	393.7 ± 250.9
CG	CG01	M	68	1.83	87.4	28	-	-	-	L	-
	CG02	M	73	1.73	82.6	28	-	-	-	L	-
	CG03	M	59	1.68	119.1	29	-	-	-	L	-
	CG04	M	72	1.73	85.3	29	-	-	-	L	-
	CG05	F	70	1.57	58.3	29	-	-	-	L	-
	CG06	M	70	1.58	58.9	29	-	-	-	L	-
	CG07	F	66	1.54	64.5	29	-	-	-	L	-
	CG08	F	72	1.52	78.1	29	-	-	-	L	-
	CG09	F	70	1.58	50.0	28	-	-	-	L	-
	CG10	F	72	1.52	54.4	27	-	-	-	L	-
	CG11	F	59	1.64	76.6	28	-	-	-	L	-
	CG12	M	61	1.67	62.2	30	-	-	-	R	-
	CG13	M	56	1.73	72.0	29	-	-	-	L	-
	CG14	F	57	1.52	61.5	28	-	-	-	L	-
	CG15	M	64	1.69	59.5	29	-	-	-	L	-
	CG16	F	69	1.55	61.0	27	-	-	-	L	-
Mean ± SD		8F/8M	66 ± 5.9	1.63 ± 0.09	70.7 ± 17.2	28.5 ± 0.8	-	-	-	1R/15 l	-

M, male; F, female; MMSE, mini-mental state examination; UPDRS, unified Parkinson’s disease rating scale; H&Y, Hoehn and Yahr; MA, most affected; NP, non-preferred; PwPD, people with Parkinson’s disease; CG, control group.

**Figure 2:**
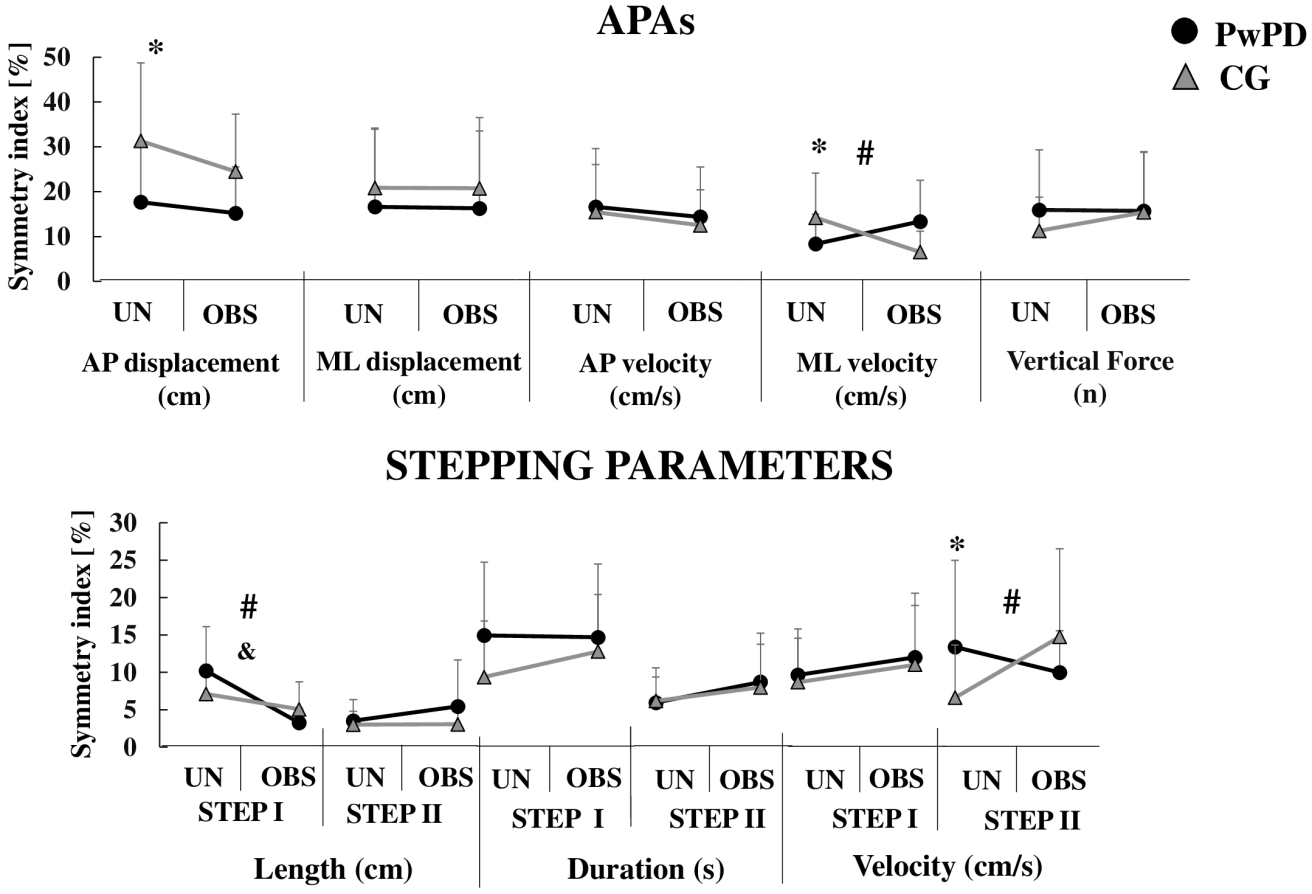
Means and standard deviations of the APAs and stepping parameters symmetry index during unobstructed (UN) and obstructed (OBS) GI in PwPD and CG. *: Significant main effect of group; &: Significant main effect of GI condition; #: Significant group by condition interaction. For clarity, the symbol for a significant main effect of group (*) is located on the left of each sub-figure, where the unobstructed data is presented.

**Figure 3:**
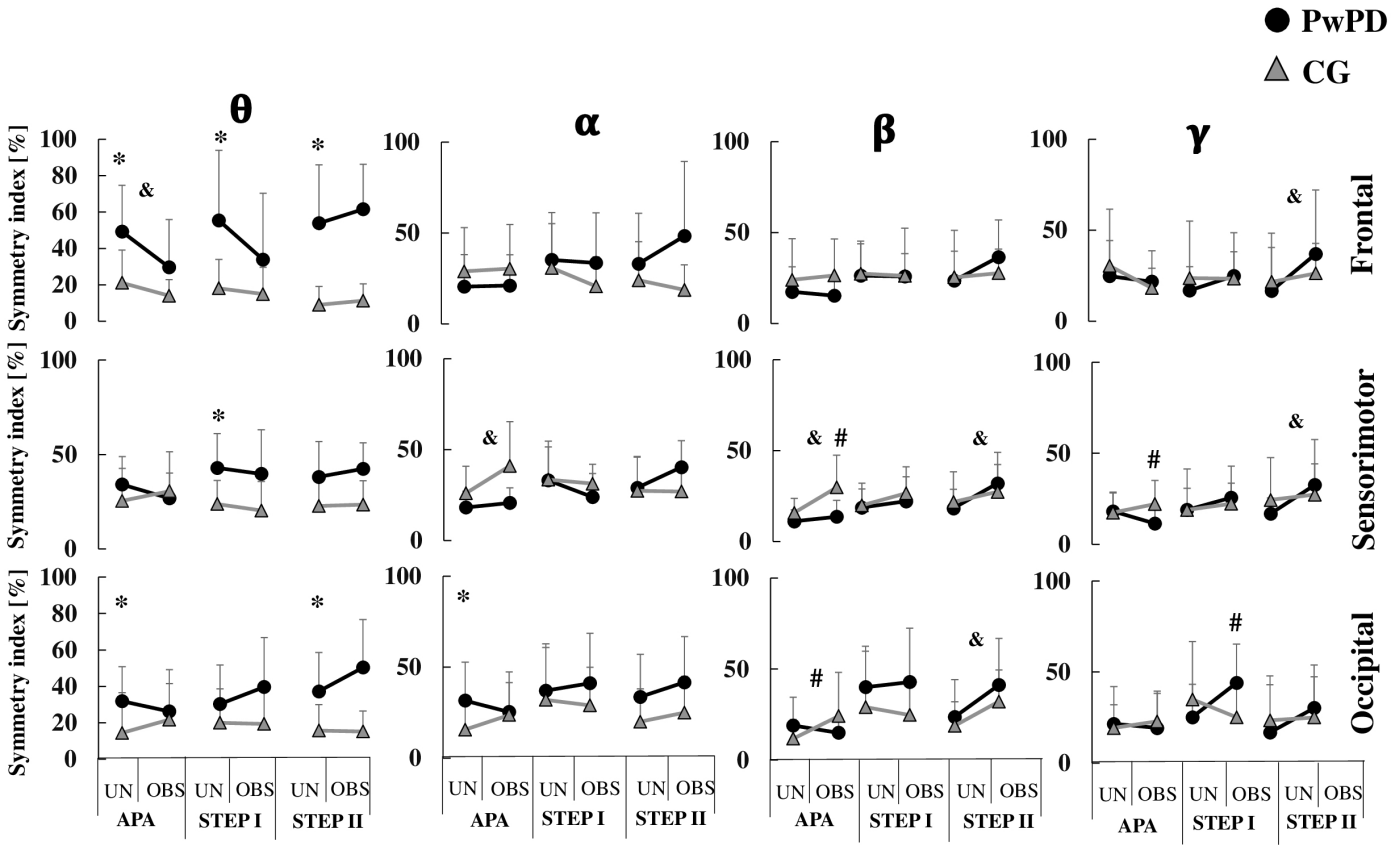
Means and standard deviations of the PSD symmetry index of theta (*θ*), alpha (*α*), beta (*β*), and gamma (*γ*) bands in each brain region (frontal, sensorimotor, and occipital areas) in the PwPD and CG. The cortical activity is presented according to APA, STEP I and II phases during unobstructed (UN) and obstructed (OBS) gait initiation. *: Significant main effect of group; &: Significant main effect of GI condition; #: Significant group by condition interaction. For clarity, the symbol for a significant main effect of group (*) is located on the left of each sub-figure, where the unobstructed data is presented.

The symmetry index is plotted in Figures 2, 3 as a function of group (PwPD vs. control) and GI condition (unobstructed vs. obstructed). The symmetry index is plotted for APAs, stepping (Figure 2) and cortical activity parameters (Figure 3). Note that the statistics to quantify symmetry during GI for PwPD included only the unobstructed GI condition (Section 3.1). For completeness, Figures 2, 3 contain both GI conditions. All statistically significant results reported below showed a *large* effect size. The means and standard deviations of the APAs, stepping and cortical activity parameters of each limb (most and least affected limb) according to GI phase and condition are presented in the supplementary material (Supplementary Tables S1–S3).

### 3.1. Effects of Parkinson’s disease on unobstructed GI asymmetry

During APA phase, the PwPD showed reduced asymmetry of anterior–posterior displacement (*F*_1,30_ = 6.55; *p* < 0.01, *η*^2^ = 0.91) and medial-lateral velocity (*F*_1,30_ = 3.83; *p* < 0.05, *η*^2^ = 0.69) vs. CG (Figure 2). However, the PwPD presented higher PSD asymmetry in the *θ* band of the frontal (*F*_1,29_ = 12.71; *p* < 0.001, *η*^2^ = 1.27) and occipital (*F*_1,29_ = 5.55; *p* < 0.02, *η*^2^ = 0.84) areas, and in the *α* band of the occipital area (*F*_1,29_ = 5.97; *p* < 0.02, *η*^2^ = 0.88; Figure 3).

During STEP I phase, no significant difference in asymmetries was observed between groups for stepping parameters (*p* > 0.05; Figure 2). The PwPD showed higher PSD asymmetry in the *θ* band of the frontal (*F*_1,29_ = 12.16; *p* < 0.002, *η*^2^ = 1.26) and sensorimotor (*F*_1,29_ = 11.44; *p* < 0.002, *η*^2^ = 1.28) areas vs. CG (Figure 3).

During STEP II phase, the PwPD showed higher asymmetry of the step velocity (*F*_1,30_ = 3.98; *p* < 0.05, *η*^2^ = 0.71; Figure 2), and *θ* band of the frontal (*F*_1,29_ = 26.8; *p* < 0.001, *η*^2^ = 1.88) and occipital (*F*_1,29_ = 10.63; *p* < 0.003, *η*^2^ = 1.18) areas vs. CG (Figure 3).

### 3.2. Impact of obstacle avoidance on GI asymmetry

There were no obstacle contacts during GI when the obstacle was present.

#### 3.2.1. APA phase

A reduced asymmetry in the frontal theta PSD (*F*_1,29_ = 7.77, *p* < 0.009, *η*^2^ = 0.21) was observed during obstructed GI vs. unobstructed GI. A higher asymmetry was observed in the *α* and *β* bands of the sensorimotor area (*F*_1,29_ = 12.16, *p* < 0.002, *η*^2^ = 0.29; *F*_1,29_ = 4.55, *p* < 0.04, *η*^2^ = 0.13, respectively) during obstructed GI (Figure 3).

A group by condition effect was observed for asymmetry of medial-lateral velocity (*F*_1,30_ = 11.91, *p* < 0.002, *η*^2^ = 0.28), *β* band of the sensorimotor (*F*_1,29_ = 5.87, *p* < 0.022, *η*^2^ = 0.16) and occipital (*F*_1,29_ = 5.79, *p* < 0.023, *η*^2^ = 0.16) areas, and *γ* band of the occipital area (*F*_1,29_ = 4.75, *p* < 0.038, *η*^2^ = 0.14) areas (Figures 2, 3). Post-hoc analyses revealed that for medial-lateral velocity asymmetry, the CG became less asymmetric when an obstacle was in place, while the PwPD became more asymmetric (*p* < 0.01). For cortical activity, the *β* band in sensorimotor (*p* < 0.003) and occipital (*p* < 0.01) areas became more asymmetrical for the CG when the obstacle was in place, but PwPD reduced PSD asymmetry in the *β* band of the sensorimotor area (*p* < 0.003) and *γ* band of the occipital area (*p* < 0.01) during obstructed GI.

#### 3.2.2. STEP I phase

A reduced step length asymmetry (*F*_1,30_ = 23.02, *p* < 0.001, *η*^2^ = 0.43) was observed in the obstructed vs. unobstructed GI (Figure 2). No significant difference was observed between conditions for cortical activity parameters (*p* > 0.05; Figure 3).

A group by condition effect was observed for asymmetry of step length (*F*_1,30_ = 6.87, *p* < 0.014, *η*^2^ = 0.18) and *γ* band of the occipital area (*F*_1,29_ = 10.12, *p* < 0.003, *η*^2^ = 0.25). Post-hoc analyses revealed that PwPD became less asymmetric for step length (*p* < 0.001) and more asymmetric for *γ* band of the occipital area (*p* < 0.005) when an obstacle was in place, but CG was not affected (Figures 2, 3).

#### 3.2.3. STEP II phase

A higher PSD asymmetry in the of the *γ* band of the frontal (*F*_1,29_ = 6.93, *p* < 0.013, *η*^2^ = 0.19) and sensorimotor (*F*_1,29_ = 4.33, *p* < 0.046, *η*^2^ = 0.13) areas and *β* band of the occipital (*F*_1,29_ = 14.35, *p* < 0.001, *η*^2^ = 0.33) and sensorimotor (*F*_1,29_ = 10.91, *p* < 0.003, *η*^2^ = 0.27) areas were found in the obstructed vs. unobstructed GI (Figures 2, 3).

A group by condition effect was observed for asymmetry of step velocity (*F*_1,30_ = 8.41, *p* < 0.007, *η*^2^ = 0.21). Post-hoc analyses revealed that CG became more asymmetric for step velocity (*p* < 0.007) when an obstacle was in place, but PwPD were not affected (Figure 2).

## 4. Discussion

Both hypotheses of our study were partially supported. As expected in the first hypothesis, PwPD showed higher asymmetry in cortical activity during APA, STEP I and STEP II phases (especially *θ* band of the frontal, sensorimotor and occipital areas) and step velocity (STEP II phase) during unobstructed GI than CG. However, unexpectedly, PwPD reduced the level of asymmetry of anterior–posterior displacement and medial-lateral velocity of the APAs. In other words, our results advanced in the literature about Parkinson’s disease asymmetry, indicating that PwPD reduced asymmetry of APAs parameters during GI, but not for cortical activity and step velocity (STEP II phase) parameters.

Regarding the second hypothesis, in general (main effect), the obstructed GI had minimal effect on the level of asymmetry in APAs parameters, reducing only the step length asymmetry during STEP I phase, and increasing cortical activity asymmetry for APA and STEP II phases (only the asymmetry of *θ* band of frontal area reduced asymmetry). Specifically for PwPD, APAs (medial-lateral velocity of CoP) became more asymmetric when an obstacle was in place, with reduced (*β* band of the sensorimotor area and *γ* band of the occipital area) and increased (*γ* band of occipital area) asymmetry of the cortical activity during APA phase and STEP I phase, respectively. Here, we show that when an obstacle was in place, the asymmetry was not regulated—either APA or cortical activity asymmetry—in PwPD.

In the next two subsections, we provide explanations about reduced APAs and increased cortical activity asymmetries in PwPD and give interpretations of obstacle influence on level of asymmetry during GI.

### 4.1. Are people with Parkinson’s disease more asymmetric than controls during gait initiation?

PwPD were not more asymmetric for APAs and most of stepping parameters of STEPS I and II phases, with the exception for step velocity of STEP II phase. Conversely, PwPD were more asymmetric for cortical activity parameters vs. CG. The reduced asymmetry in APAs indicated that PwPD may use similar postural adjustments to prepare for GI, independent of the initiating limb. Reduced asymmetry in PwPD is contrary to the effects with other populations that were asymmetry during GI: hemiparetic patients (Hesse et al., 1997), individuals with severe unilateral knee arthritis (Viton et al., 2000) and hemiplegic patients (Bensoussan et al., 2006). Also, our findings are contrary to previous studies that analyzed level walking and showed higher asymmetry in PwPD compared to their peers (Baltadjieva et al., 2006; Plotnik and Hausdorff, 2008; Roemmich et al., 2014; Fling et al., 2018). We can explain this inconsistency from two perspectives. First, literature is consistent to show that PwPD use a rigid strategy, maintaining similar motor behavior among trials, when the task is not challenging, avoiding unbalance and extra mechanical cost and prioritizing safety (Yogev-Seligmann et al., 2012). Possibly, using comparable APAs adjustments when initiate the gait with most and least affected limb may be a cautious strategy considering that no individual presented falls, slips or freezing episodes during GI in this study. On the other hand, we have included in the study a sample of people with mild to moderate stage of Parkinson’s disease without freezing of gait history, which may not affect GI and allows the participants to maintain a similar behavior of APAs. However, we should not interpret that this symmetry behavior is good for GI, considering that PwPD showed slower movements and smaller CoP displacements during GI when compared to CG (Rogers et al., 2011; Delval et al., 2014; Boonstra et al., 2016; Simieli, 2021, under review, see footnote 1), which impaired GI in this population.

The second explanation for the inconsistent change in asymmetry across populations is related with dopaminergic system impairments. The neurodegeneration of dopaminergic neurons, especially the substantia nigra pars compacta, inhibits the thalamocortical and brainstem motor systems (Obeso et al., 2008), leading to impairments on motor adjustments during GI (Delval et al., 2014; Simieli, 2021, under review, see footnote 1). In addition, the neural neurodegeneration is asymmetric in Parkinson’s disease (Riederer and Sian-Hülsmann, 2012; Claassen et al., 2016), which induces functional asymmetry between hemispheres, with a relative reduction of neural excitability on the most affected hemisphere and a supposed increase on the least affected one. Also, PwPD present reduced transcallosal sensorimotor structural connectivity (Fling et al., 2018). Transcallosal pathways has an important function to balance mutual inhibitory control in the motor cortex (Fling et al., 2011). With Parkinson’s disease, this balance of transcallosal inhibitory circuits between the motor areas in both hemispheres becomes unequal (Fling et al., 2018), which may be a significant mechanism underlying asymmetries in cortical activity. A higher asymmetry in cortical activity parameters in PwPD, as showed in all GI phases (APA, STEP I, and STEP II phases), specially the asymmetry in the frontal region (Little et al., 2012; Mostile et al., 2015), may lead to bradykinesia and akinesia symptoms during movement. Slowness and much smaller movement symptoms may inhibit postural adjustments, explaining no-change in APAs during GI. Also, we should consider that our study was conducted with PwPD in ON medication state. Previous studies have shown that levodopa modifies asymmetry, improves APAs (Plotnik et al., 2005; Frazzitta et al., 2013) and impacts the estimates of PSD (Brown and Marsden, 1999; Li et al., 2016; Chung et al., 2018). Thus, any discussion related to the pathophysiology of Parkinson’s disease should be considered carefully.

The asymmetric cortical activity behavior during GI in PwPD may be considered between adaptive or maladaptive change of brain control (Peterson and Fling, 2018; Berenguer-Rocha et al., 2020; Knights et al., 2021), which was also reported in stroke studies (Berenguer-Rocha et al., 2020). One may argue that cortical activity asymmetry is an adaptive change because damaged areas of most affected hemisphere are substituted by residual networks within both hemispheres (Peterson and Fling, 2018; Berenguer-Rocha et al., 2020; Hill et al., 2020; Knights et al., 2021). The remapping of functional representation from degenerated areas onto homologous areas within the least-degeneration hemisphere causes an increased activity in the least affected motor cortex (Berenguer-Rocha et al., 2020) and a greater asymmetry in cortical activity. On the other hand, another may consider that cortical activity asymmetry is a maladaptive change due to greater neural and/or hemodynamic activity for the same computation, causing a neural inefficiency (Peterson and Fling, 2018; Berenguer-Rocha et al., 2020; Hill et al., 2020; Knights et al., 2021). Alternatively, there is growing evidence of neural dedifferentiation (Hill et al., 2020; Knights et al., 2021), whereby the functional specificity of brain regions reduces with age and degeneration, but requiring additional ipsilateral areas involvement in tasks that were not required when younger (Knights et al., 2021).

If PwPD with lower motor asymmetry during GI had higher asymmetry in cortical activity, this would provide support of an adaptive mechanism to improve motor behavior (GI). Considering that an asymmetry in APAs and stepping parameters during GI was expected in PwPD due to impairments in the dopaminergic system (Obeso et al., 2008; Riederer and Sian-Hülsmann, 2012; Claassen et al., 2016), we are more likely to interpret this asymmetric cortical activity behavior as an adaptive change, considering that the PwPD were effective during GI with both limbs, reducing motor asymmetry. However, this is the first study that investigate the asymmetry in cortical activity during GI in PwPD, consequently, more studies are necessary to confirm our supposition in favor of adaptive change in cortical activity. Our findings advance in the understating underlying of (As) symmetry in motor and cortical activity during GI in PwPD.

The only stepping parameter more asymmetric for PwPD was step velocity during STEP II. This is not an easy finding to explain. We may suggest that when most affected limb was the trailing limb during first step of GI, PwPD need to increase the angular momentum of trailing limb, especially around ankle joint, during the second step. Considering that most affected limb showed higher step velocity (57.77 ± 7.26 cm/s) vs. least affected limb (52.69 ± 7.32 cm/s) in the second step and the exacerbated bradicinesia and akinesia in the most affected side (Greenland and Barker, 2018), we can suggest that GI leading with most affected limb requires an increasing in angular acceleration to move the limb in the second step. This is a supposing since we did not measure angular acceleration in this study. Another possible explanation is that when PwPD initiate the gait (STEP I) using the least affected limb, they have a more balanced, more controlled, and better first step. Performing a better first step helps to improve the performance for the next step (STEP II), even though it was the impaired limb. Conversely, the relatively poor first step in the other GI condition means that the second step is also poor. However, an asymmetry in the first step would expect to see as well, which does not happen.

### 4.2. The presence of obstacle during gait initiation does not regulate motor asymmetry in Parkinson’s disease

Parkinson’s disease only reduced motor asymmetry in the obstructed GI of the step length during STEP I. This finding suggests that obstructed GI did not regulate motor asymmetry in PwPD, contrariwise, the asymmetry of the medial-lateral velocity of CoP increased. We can interpret that the presence of obstacle provided a visual cue for both GI with most and least affected limb, similar to our previous study (Simieli, 2021, under review, see footnote 1), guiding the GI and improving the cortical activity (Beeler, 2011; Beeler et al., 2013). The presence of an obstacle seems to optimize cognitive resources in both condition of GI, with most and least affected limbs, only presenting higher asymmetry in cortical activity on occipital area during STEP I phase in PwPD. According to our explanation above, we can understand this behavior as an adaptive change, which results in no motor asymmetry and changing in the level of asymmetry in cortical activity. Also, a prominent lateralization (asymmetry) of activity in occipital region may represent a greater response to dopaminergic stimulation (Mostile et al., 2015), helping to bypass the impaired basal ganglia and reducing motor asymmetry during GI. On the other hand, we also need to consider that the obstacle height may not be enough to facilitate a compensatory shift to goal-directed control of movement, and consequently, to reduce motor asymmetry when compared to unobstructed GI.

An interesting finding of this study was the reduction of asymmetry in the medial-lateral velocity of CoP with an increased asymmetry in the *β* band of the sensorimotor and occipital areas during obstructed GI in CG. An asymmetry in these areas indicates rapid desynchronization and subsequent resynchronization of *β* activity (called post-motion beta rebound; Gaetz et al., 2010; Heinrichs-Graham et al., 2014b). This event is associated with movement planning/selection (Heinrichs-Graham and Wilson, 2015, 2016) that may involve in the reduction of motor asymmetry during GI. This behavior seems an adaptive change regulating GI asymmetry in CG.

## 5. Conclusion and limitations

PwPD were not motor asymmetric (APAs and stepping parameters) during GI, but they presented a higher cortical activity asymmetry compared to CG. These changes in cortical activity can be interpreted as an adaptive behavior to reduce motor asymmetry during GI. In addition, the presence of obstacle did not regulate motor asymmetry during GI, but the obstacle reduced the level of asymmetry in cortical activity compared to unobstructed GI in PwPD.

Despite the observation that PwPD had no increased asymmetry during GI (unobstructed and obstructed conditions), our study did not answer if GI using least affected limb is beneficial or not for PwPD. We did not address the motor adjustments and cortical activity (only asymmetry) changes when the gait was initiated with one or another limb, which have likely practical applications in clinical practice and should be answered in future studies. In addition, the instruction about which foot should initiate the walking may influence the planning and adjustments of the participants. However, it is difficult to design a protocol for analyzing asymmetry without this instruction. Moreover, we did not analyze gaze behavior in this study. So, the consideration of obstacle was a visual cue should be interpreted with caution. In addition, the stage of disease, side most affected and subtypes (tremor at rest or postural instability) of Parkinson’s disease may be a confounding factor considering that we have included people in unilateral and bilateral stages of disease, right and left most affected limb, and individuals with different subtypes in the study. Finally, despite the literature has no determined a minimum number of trials to analyze the cortical activity EEG-PSD in walking and GI protocols and similar previous studies with walking and people with PD used similar number of trials for the PSD analysis (Orcioli-Silva et al., 2021), one may argue that the number of trials of GI performed to represent cortical activity through EEG-PSD analysis was low.

## Data availability statement

The original contributions presented in the study are included in the article/Supplementary material, further inquiries can be directed to the corresponding author.

## Ethics statement

The studies involving human participants were reviewed and approved by Ethical Committee of School of Science at UNESP (CAAE #56031316.9.0000.5398). The patients/participants provided their written informed consent to participate in this study.

## Author contributions

MF, LS, SR, and FB conceptualized and designed the studies. MF, LS, and FB performed the data acquisition and wrote the manuscript. SR, TP, and FS provided technical support at every step of the manuscript and technical analyses. MF, SR, and FB interpreted the results. All authors contributed to the article and approved the submitted version.

## Funding

This study was financially supported by São Paulo Research Foundation (FAPESP—#2018/21870–7; #2019/24752–8; #2016/14950–6; and #2022/02971-2) and partly supported by the Coordenação de Aperfeiçoamento de Pessoal de Nível Superior—Brasil (CAPES)—Finance Code 001.

## Conflict of interest

The authors declare that the research was conducted in the absence of any commercial or financial relationships that could be construed as a potential conflict of interest.

## Publisher’s note

All claims expressed in this article are solely those of the authors and do not necessarily represent those of their affiliated organizations, or those of the publisher, the editors and the reviewers. Any product that may be evaluated in this article, or claim that may be made by its manufacturer, is not guaranteed or endorsed by the publisher.

## Supplementary material

The Supplementary material for this article can be found online at: https://www.frontiersin.org/articles/10.3389/fnagi.2023.1142540/full#supplementary-material

## Footnotes

^1^Simieli, L. (2021). Presence of an obstacle during gait initiation does not affect anticipatory postural adjustments but does affect spatial-temporal parameters in people with Parkinson’s disease. Gait Posture 22 (Under review).

## Abbreviations

PwPD: People with Parkinson’s disease
GI: Gait initiation
APAs: Anticipatory postural adjustments
GC: Control group
CoP: Center of pressure
