# Supplementary material for: (A)symmetry during gait initiation in people with Parkinson’s disease: A motor and cortical activity exploratory study
Source: Front Aging Neurosci. 2023 Apr 17;15:1142540. doi: 10.3389/fnagi.2023.1142540 (PMC10150081; doi:10.3389/fnagi.2023.1142540)
Supplement: Supplementary file 2 [file Table_2.docx]

**Table S2.** Means and standard deviations of the stepping parameters during unobstructed and obstructed GI in PwPD and CG.
