# Supplementary material for: (A)symmetry during gait initiation in people with Parkinson’s disease: A motor and cortical activity exploratory study
Source: Front Aging Neurosci. 2023 Apr 17;15:1142540. doi: 10.3389/fnagi.2023.1142540 (PMC10150081; doi:10.3389/fnagi.2023.1142540)
Supplement: Supplementary file 3 [file Table_3.docx]

**TABLE S3.** Means and standard deviations of the PSD data of θ, α, β and γ bands in each brain region (frontal, sensorimotor, and occipital areas) in the PwPD and CG. The cortical activity is presented according to APA, STEP I and II phases during unobstructed and obstructed GI.
